# Sensory Analysis of Cupuaçu (*Theobroma grandiflorum*) Melomel Produced with *Saccharomyces cerevisiae* var. *boulardii* Using Acceptance Testing and the Check-All-That-Apply (CATA) Method

**DOI:** 10.3390/foods15193411

**Published:** 2026-09-24

**Authors:** Karina Nascimento Pereira, Handray Fernandes de Souza, Ricardo Donizete Teixeira, Amanda Cristina Dias de Oliveira, Victor Dédalo Di Prospero, Rafael Lopes de Alcântara, Marina Vieira de Carvalho, Carlos Augusto Fernandes de Oliveira, Eliana Setsuko Kamimura

**Affiliations:** Department of Food Engineering, School of Animal Science and Food Engineering, University of São Paulo, Av. Duque de Caxias Norte, 225, Pirassununga 13635-900, SP, Brazil; ricardo.donizete@usp.br (R.D.T.); amandacdo@usp.br (A.C.D.d.O.); victordedalo@usp.br (V.D.D.P.); rafaellopesdealcantara@usp.br (R.L.d.A.); carvalhomv@usp.br (M.V.d.C.); carlosaf@usp.br (C.A.F.d.O.); elianask@usp.br (E.S.K.)

**Keywords:** beverages, alcoholic fermentation, consumer acceptance, probiotic yeast

## Abstract

Mead is a fermented beverage produced through the yeast fermentation of diluted honey. Among its variations, melomel stands out as it is prepared with the addition of fruits to enhance the sensory and nutritional profile of the beverage. Although *Saccharomyces cerevisiae* is the conventional choice for mead fermentation, the probiotic yeast *Saccharomyces cerevisiae var. boulardii* has emerged as a promising starter culture to develop functional alcoholic beverages. This study aimed to evaluate the sensory profile of a cupuaçu (*Theobroma grandiflorum*) melomel fermented with *S. boulardii* using the consumers acceptance and the Check-All-That-Apply (CATA) methodologies. Accordingly, two treatments were designed for the sensory analysis: (i) Treatment 1 (T1), consisting of cupuaçu melomel fermented with the probiotic yeast *S. boulardii* at a concentration of 1 g/L; and (ii) Treatment 2 (T2), consisting of cupuaçu melomel fermented with the commercial yeast *S. cerevisiae*, also at a concentration of 1 g/L. The products were evaluated by 113 healthy volunteer consumers, aged 18 years or older, who were not pregnant and had no history of allergy or intolerance to alcohol, honey, and/or cupuaçu. Although the participants had limited familiarity with honey wine, all reported being familiar with and enjoying both honey and cupuaçu. Compared with the cupuaçu melomel fermented with the conventional yeast *S. cerevisiae*, the melomel fermented with *S. boulardii* achieved higher scores (*p* < 0.05) for flavor, overall impression and purchase intent. CATA analysis revealed that attributes indicating “light amber color,” “clear,” “honey aroma,” “sweet taste,” “honey flavor,” and “Flavorful” were associated with the *S. boulardii* formulation. In conclusion, *S. boulardii* represents a viable alternative culture for melomel processing, supporting the development of fermented beverages with improved sensory characteristics and good consumer acceptance.

## 1. Introduction

Mead is a traditional alcoholic beverage produced through the yeast fermentation of a diluted honey solution. In Brazil, [1] this beverage usually contains an alcohol content between 4 and 14% (*v/v*) at 20 °C, [2] although recent studies reported meads with ethanol concentrations ranging from 8 to 18% (*v/v*), depending on fermentation conditions, must composition, and the yeast strain utilized [3,4]. Its production and consumption date back to antiquity, being considered one of the oldest fermented beverages known to humanity [2]. Over the years, its basic formulation has undergone several adaptations through the addition of herbs, spices, vegetables, and especially regional and seasonal fruits, resulting in a new category of mead known as melomel [5]. The chemical composition and sensory attributes of this beverage are directly influenced by the type of honey used, the yeast employed, the fermentation conditions, and the added ingredients [6]. In this context, the incorporation of fruits resulting in melomel contributes to improving the beverage by adding distinctive sensory characteristics and chemical compounds that may enhance its quality and complexity. Among the fruits used in melomel production, cupuaçu (*Theobroma grandiflorum*) stands out as a native Amazonian fruit widely produced in the Brazilian Amazon region. It is characterized by its yellowish color, intense and acidic flavor, and pleasant, distinctive aroma. Due to these sensory characteristics and its high acceptance among consumers, cupuaçu pulp is widely used in the production of beverages, sweets, and frozen desserts [7]. In addition to its sensory attributes, fruits of the *Theobroma* genus, such as cupuaçu pulp, are sources of fiber, polyphenols, essential amino acids, and minerals [8].

*Saccharomyces cerevisiae* (*S. cerevisiae*) is one of the most widely used yeasts in the production of fermented alcoholic beverages, including mead and melomel. However, the application of the probiotic yeast *Saccharomyces cerevisiae* var. *boulardii (S. boulardii*) in the production of different alcoholic fermented beverages has been increasingly reported in the literature [9,10]. Its ethanol production capacity and cell viability after the fermentation process enable the development of beverages with both alcoholic and probiotic characteristics [10,11]. Recently, Pereira et al., (2025) [4] demonstrated the viability of producing a cupuaçu melomel using *Saccharomyces cerevisiae* var*. boulardii* as the fermenting microorganism. The study showed the yeast’s potential to provide probiotic properties to the beverage, adding functional value to the product [4]. In addition, the survival of *S. boulardii* was observed under simulated gastrointestinal tract conditions, which is a key characteristic for its action as a probiotic.

Although several factors influence the consumption of alcoholic beverages, flavor is one of the main determinants of sensory acceptance. In this context, sensory evaluation plays an important role in quality control, as well as in the development and safety assessment of new food products [12]. The use of a central composite design allowed the optimization of fermentation conditions to obtain a beverage with adequate physicochemical characteristics. However, the sensory properties of the produced melomel have not yet been evaluated, representing an important knowledge gap and an opportunity for future research. In this context, this study aimed to characterize the sensory acceptance of cupuaçu melomel made with the probiotic yeast *Saccharomyces cerevisiae* var*. boulardii*, using acceptance tests and the Check-All-That-Apply (CATA) methodology.

## 2. Materials and Methods

### 2.1. Microorganisms

*S. boulardii* CCT 4308 (UFPEDA 1176) strain was obtained from the culture bank of the André Tosello Foundation (FAT, research and technology, Brazil) in Campinas, São Paulo, Brazil. For use, the strains were cultured in 250 mL Erlenmeyer flasks containing YPD medium (yeast extract, peptone, and dextrose) under agitation in an incubator (Shaker incubator, TECNAL, model TE-424, Niort, France) at 160 rpm and 25 °C for 96 h. After growth, the cells were harvested by centrifugation, washed three times with phosphate buffer at pH 7.2, divided into portions, and used for inoculation into the mead wort [4]. To produce the beverage, the wet weight of *S. boulardii* was used at a concentration of 1 g/L of must, corresponding to 10^6^ CFU/mL [4].

Commercial *S. cerevisiae* yeast (Red Star, Premier Rouge, Fermentis Lesaffre for Beverages) was obtained from a local supplier. The yeast was weighed according to the manufacturer’s recommendations and then hydrated in 50 mL of melomel wort. To produce the beverage, the wet weight of *S. cerevisiae* was used at a concentration of 1 g/L of wort, corresponding to 10^7^ CFU/mL.

### 2.2. Cupuaçu Melomel Preparation

To conduct the sensory analysis, the production of cupuaçu mead followed the standardized procedure and methodology described by Pereira et al. (2025) [4], with the preparation of new batches of the beverage. Two treatments were prepared: (i) Treatment 1 (T1), corresponding to cupuaçu melomel fermented with the probiotic yeast *S. boulardii* at a concentration of 1 g/L; and (ii) Treatment 2 (T2), consisting of cupuaçu mead fermented with the commercial yeast *S. cerevisiae*, also at a concentration of 1 g/L.

In general, the musts for the cupuaçu mead were prepared separately. The honey was first diluted in drinking water according to previous calculations by Pereira et al. (2025) [4], while the cupuaçu pulp was liquefied in a 1:10 (*w*/*v*) ratio, using 100 g (10%) of pulp in 1000 mL of drinking water. Both components were then pasteurized at 65 °C for 30 min and mixed in a ratio of 80% honey must to 20% cupuaçu juice. After pasteurization, the mixture was cooled to 25 °C.

Next, approximately 4 L of pasteurized wort was transferred to 5 L polypropylene fermentation buckets. A yeast concentration of 1 g/L was then added to the wort. Subsequently, the fermentation buckets were sealed with lids equipped with airlocks to maintain anaerobic conditions inside. The fermentation buckets were incubated in a BOD incubator (model 347 CD, São Paulo, Brazil, MERSE), and fermentation took place over 10 days at a temperature of 25 °C.

It should be noted that, for the production of cupuaçu mead, three replicates were conducted for each treatment, totaling six independent fermentation buckets. Given that the preparation of the mead followed standardized procedures and methodology, as described by Pereira et al. (2025) [4], no physicochemical analyses or viable yeast cell counts were performed in the present study, since these parameters had already been previously evaluated and reported by those authors. Thus, the beverages were produced with the specific objective of evaluating their sensory characteristics and determining consumer acceptance of the products.

### 2.3. Microbiological Analyses for Total Coliforms, Escherichia coli and Salmonella *spp.*

To ensure the hygienic and sanitary quality of the melomel for sensory analysis, microbiological analyses were performed to detect contaminating microorganisms, including total coliforms (TC), *Escherichia coli*, and *Salmonella* spp., according to the methodology described by [13]. *E. coli* and TC analyses were carried out using ready-to-use CompactDry™ EC plates (R-Biopharm AG, Compact Dry EC, Tokyo, Japan), as recommended by the manufacturer. Samples were first homogenized and serially diluted in sterile 0.9% saline peptone water to obtain 10^−2^ and 10^−3^ dilutions. Next, 1 mL of both the undiluted sample and each dilution was aseptically inoculated onto the center of the CompactDry™ EC plates, where the absorbent medium automatically and evenly distributed the sample across the surface. Inoculated plates were incubated at 37 ± 1 °C for 24 ± 2 h. Following incubation, colonies were enumerated based on their distinctive coloration in the chromogenic medium: *E. coli* colonies appeared blue-to-purple, whereas TC appeared red-to-pink. The TC count was calculated by adding the red/pink and blue/purple colonies, with results expressed in colony-forming units (CFU)/mL.

For *Salmonella* spp. detection, CompactDry™ SL kits (R-Biopharm AG, Compact Dry SL, Tokyo, Japan) were employed. First, 25 mL samples underwent a pre-enrichment step in Lactate Ringer’s Broth (LRB) (KASVI, K25-1206, Madrid, Spain) to recover injured cells and enhance analytical sensitivity. These mixtures were incubated at 35 ± 2 °C for 24 ± 2 h under aerobic conditions. After pre-enrichment, a 1 mL aliquot of each sample was aseptically inoculated onto the center of the CompactDry™ SL plates for automatic and uniform distribution. The plates were incubated according to the manufacturer’s guidelines. Presumptive *Salmonella* spp. colonies were identified by their typical chromogenic profile (black/green, blue/purple, or yellow, occurring individually or mixed). All microbiological assays were performed in independent triplicates to ensure data reliability and reproducibility.

### 2.4. Sensory Analysis

The study was previously approved by the Human Research Ethics Committee of the University of São Paulo (USP), under CAAE protocol No. 69006423.6.0000.54220. Healthy volunteers aged 18 years or older (the legal drinking age in Brazil), who were not pregnant and had no history of allergy or intolerance to alcohol, honey, and/or cupuaçu, participated in the study. Before testing, all participants were briefed on the study’s objectives, procedures, and potential risks. The volunteers then signed an Informed Consent Form (ICF), which guaranteed that their participation was completely voluntary and that they could withdraw from the study at any time without penalty. Cupuaçu melomel samples were served at 4 ± 1 °C in individual booths under white lighting conditions. Approximately 20 mL of each beverage was offered in 30 mL acrylic cups, randomly labeled with three-digit codes. The samples were presented to consumers in random order to minimize potential order effects. Water and unsalted crackers were provided between samples as palate cleansers to minimize sensory fatigue and potential carryover effects.

At this stage, the sensory analysis was conducted in two phases. Initially, the volunteers received the samples along with a questionnaire regarding their consumption habits of melomel, honey, and cupuaçu. Next, the sensory acceptability of the beverages was evaluated, considering the attributes of appearance, aroma, color, flavor, and overall impression. These parameters were evaluated using a structured nine-point hedonic scale, ranging from 1 (“dislike extremely”) to 9 (“like extremely”). Subsequently, the purchase intent test was applied using a structured seven-point categorical scale, ranging from 1 (“I would never buy it”) to 7 (“I would always buy it”), to assess the product’s potential for commercial acceptance among consumers.

It should be noted that, for the sensory analysis, the sample size was not determined through a formal a priori statistical power calculation. Instead, the target number of participants was established based on methodological recommendations and standard practice in quantitative sensory analysis studies with consumers, taking into account the exploratory and comparative objectives of this study. Thus, recommendations in the sensory analysis literature indicate that approximately 100 consumers are commonly considered adequate for small-scale quantitative consumer tests, while tests conducted at centralized sites often involve 100 or more responses per product [14]. In light of this, a total of 113 consumers were included in this study.

#### Check-All-That-Apply (CATA) Method

Panelists received a sensory evaluation form based on the Check-All-That-Apply (CATA) method. A list of attributes previously defined by the research team was provided, including sensory attributes selected from previously published studies that conducted sensory evaluations of mead and melomel [2,9,15]. The participants were instructed to select all terms they considered appropriate to describe each of the evaluated samples.

The attributes presented to the consumers included visual, aroma, and taste characteristics, namely: yellowish color, light amber color, clear, cloudy, fruity aroma, honey aroma, cupuaçu aroma, alcoholic aroma, vinegary aroma, sweet taste, bitter taste, acidity taste, cupuaçu flavor, honey flavor, dry taste, vinegary flavor, full-bodied, flavorful, distinctive, and alcoholic taste.

### 2.5. Statistical Analysis

The acceptance data were analyzed using a two-way ANOVA without replication, with the 113 consumers treated as blocks and the melomel formulations as the factor of interest. Since all consumers evaluated both formulations, the design was complete and balanced, allowing for the control of inter-individual variability in hedonic responses. This approach is consistent with the principles of design and analysis of sensory tests described by [16]. For two formulations, the blocked ANOVA is mathematically equivalent to the paired t-test, yielding the relationship (F = t^2^) and, therefore, the same statistical inference. Accordingly, the results were analyzed using analysis of variance (ANOVA), and the means were compared at a significance level of 5% (*p* < 0.05). The Check-All-That-Apply (CATA) assessment data were analyzed using Cochran’s Q test, applied individually for each sensory descriptor, with significant differences considered when *p* < 0.05 [17]. Since only two samples were evaluated, Cochran’s Q test is equivalent to the McNemar test [18]. The frequencies of the descriptors were expressed as the absolute number of mentions and as a percentage of consumers. Statistical analyses were performed using Microsoft Excel^®^ (Microsoft Corporation, Redmond, WA, USA) and RStudio^®^ version 2025.05.0+496 (Posit Software, PBC, Boston, MA, USA).

## 3. Results and Discussion

### 3.1. Microbiological Analyses of Melomel

Microbiological analysis of cupuaçu mead indicated that total coliforms, *E. coli*, and *Salmonella* spp. were not detected in the samples within the analytical limits of the methods used. Given the absence of specific microbiological standards for melomel as an independent category, these results were interpreted in light of the general hygiene requirements and good manufacturing practices applicable to foods intended for human consumption, as well as the principles established by RDC No. 724/2022 [19] and IN No. 161/2022 [20]. Thus, the samples exhibited satisfactory microbiological conditions under the evaluated conditions and analytical limits and were considered suitable for human consumption under the tested conditions.

### 3.2. Sensory Analysis

#### 3.2.1. Consumer Profile

A total of 113 consumers participated in this study, mostly female (66.4%), while male participants represented 33.6% of the sample. Table 1 presents the demographic characteristics of the participants, as well as the frequency of alcohol consumption among the consumers who participated in the sensory evaluation of the cupuaçu melomel. Regarding the age group of the cupuaçu melomel tasters, it was observed that 72.6% of the participants were between 18 and 24 years old, followed by the age groups 25–29 years (15.0%), 30–39 years (6.2%), 40–49 years (4.4%), and 50–59 years (1.8%). Concerning the frequency of alcoholic beverage consumption, most participants reported consuming alcohol once a month (36.3%) or twice a week (34.5%), followed by 21.2% who stated that they consumed alcoholic beverages occasionally. Only 0.9% of consumers reported alcohol consumption four times a week, and no participant declared daily consumption. In addition, 7.1% reported not consuming alcoholic beverages. Overall, these results indicate that the sensory panel was predominantly composed of young women with moderate or occasional alcohol consumption habits, representing a potentially relevant consumer profile for the evaluation of the sensory acceptance of cupuaçu melomel.

Figure 1 illustrates the consumers’ familiarity with melomel and their knowledge regarding honey and cupuaçu. A low level of familiarity with melomel was observed, as the majority of participants (approximately 88.5%) had no prior experience with this beverage. Notably, the 18–24 age group accounted for over 70% of the panelists; this is relevant since young adults generally exhibit greater openness to novel products [18]. Nevertheless, both honey and cupuaçu were well-known to most tasters, who reported liking these ingredients. This indicates that these raw materials hold favorable potential for the development and commercialization of cupuaçu melomel, even among consumers unfamiliar with the beverage itself.

#### 3.2.2. Product Acceptance and Purchase Intention

Table 2 presents the results of the sensory acceptance and purchase intent of the cupuaçu melomel produced with *S. boulardii* and *S. cerevisiae*. For the “overall impression” attribute, the melomel fermented with *S. boulardii* showed a significantly higher score (*p* < 0.05) than the beverage produced with *S. cerevisiae* (7.51 ± 1.15 and 7.25 ± 1.18, respectively), indicating greater overall acceptance by consumers. In this regard, it was observed that the formulation containing *S. boulardii* had an overall rating that was 0.26 points higher, corresponding to approximately 2.89% of the total scale. A similar result was observed for the “flavor” attribute, where the sample fermented with the probiotic yeast achieved a higher acceptance score (7.64 ± 1.30) compared to the one produced with *S. cerevisiae* (7.04 ± 1.52). Furthermore, it was observed that the formulation containing *S. boulardii* had a flavor rating that was 0.60 points higher, corresponding to approximately 6.67% of the total scale. Given that taste is one of the main factors determining preference and acceptance of fermented beverages, these results indicate that the melomel produced with *S. boulardii* was well accepted in terms of taste, standing out positively among the formulations evaluated.

Although no significant differences (*p* > 0.05) were observed among the treatments in terms of color, aroma, and appearance in the hedonic test, this result should be interpreted in terms of consumer acceptance, rather than as evidence that the samples were perceptually identical. Thus, the probiotic yeast caused subtle changes in flavor characteristics and overall impression that were perceptible to consumers but insufficient to significantly affect their overall acceptance based on color, aroma, or appearance. The search for strategies to improve the sensory properties of meads and melomels has been widely reported in the scientific literature, especially through the incorporation of fruits, plant extracts, and different fermentative cultures. Recent studies show that adding plant raw materials can increase the sensory complexity and consumer acceptance of these products [4,9,21]. In this context, [22] found that meads made with *Hibiscus sabdariffa* and birch sap presented superior sensory attributes compared to their respective controls. Similarly, Santos da Silva et al. (2025) [21] reported that jenipapo (*Genipa americana* L.) mead fermented with Mangrove Jack’s M05 yeast achieved higher scores for flavor, overall acceptability, and purchase intent compared to traditional mead. These studies reinforce that both the composition of the fermentative matrix and the choice of microorganism play a decisive role in the sensory quality and acceptance of honey-based fermented beverages.

Regarding purchase intent, the melomel produced with *S. boulardii* showed a significantly higher score (*p* < 0.05) than the beverage fermented with *S. cerevisiae* (4.63 ± 1.39 and 4.09 ± 1.46, respectively). The formulation containing *S. boulardii* showed a purchase intention that was 0.54 points higher, corresponding to approximately 7.7% of the total scale. This result is particularly relevant from a marketing perspective, as purchase intent is widely used as a predictive indicator of the commercial acceptance of new products. In general, higher purchase intent scores reflect a greater consumer willingness to buy and consume a product, while lower scores indicate a lower probability of market adoption [2,13]. Furthermore, these results corroborate previous studies that demonstrated higher acceptability and purchase intent for meads produced with *S. boulardii* compared to those made with commercial *S. cerevisiae* yeasts [23,24].

In light of the above, the results regarding sensory acceptance and purchase intention indicated that the use of *S. boulardii* as a fermentation culture contributed positively to the acceptance of cupuaçu mead, resulting in significant improvements in the attributes of flavor and overall impression, as well as in purchase intention.

#### 3.2.3. Check-All-That-Apply Test Results

The results of CATA sensory analysis applied to the cupuaçu melomels produced with the probiotic yeast *S. boulardii* and the commercial yeast *S. cerevisiae* are presented in Table 3. Significant differences were found between samples for several sensory attributes related to appearance, aroma, taste, and overall perception of the beverage, showing that the melomels produced with the two yeast strains differed significantly in their sensory characterization.

Regarding the visual attributes, the melomel fermented with *S. boulardii* was more frequently associated with “light amber color” and “clear,” suggesting a visually clearer beverage with a lighter amber hue. On the other hand, the sample produced with *S. cerevisiae* showed a higher association with the attribute “cloudy,” indicating a higher perception of turbidity by the evaluators. The higher frequency of the “clear” descriptor for the *S. boulardii* sample may reflect differences in clarification, suspended particles, or yeast sedimentation associated with the fermentation process; however, because instrumental turbidity measurements were not performed on the sensory batches, this interpretation should be considered exploratory. On the other hand, the attribute “yellowish color” did not differ significantly between the samples (*p* > 0.05), demonstrating that both presented similar coloration in this aspect.

However, as for taste attributes, the melomel fermented with *S. boulardii* exhibited a significantly higher association with “sweet taste”, “honey aroma”, “honey flavor”, and “flavorful”, indicating greater sweetness, flavor intensity, and typical honey notes. In contrast, the *S. cerevisiae* beverage was more frequently linked to “bitter taste”, “dry taste”, “vinegary taste” and “alcoholic taste”, suggesting sensory features that are potentially less favorable for product acceptance. The “acidity taste”, “cupuaçu flavor” and “cupuaçu aroma” showed no significant differences between treatments (*p* > 0.05), reflecting similar consumer perceptions.

For the global attributes “full-bodied” and “distinctive”, no significant differences were observed between treatments (*p* > 0.05), suggesting that the yeast type did not substantially influence these sensory features. Overall, the CATA test results indicate that the melomel fermented with *S. boulardii* presented a sensory profile more closely associated with positive attributes, including higher visual clarity, a more pronounced honey aroma, and taste characteristics related to sweetness, honey flavor, and greater sensory intensity. The methodology followed the procedure described by Pereira et al. (2025) [4], who reported statistically different soluble solids contents (°Brix) between the *S. boulardii* and *S. cerevisiae* formulations, with the *S. boulardii* formulation showing higher values. However, the values found for total titratable acidity showed no statistically significant difference between treatments. On the other hand, the higher frequency of the “sweet taste” descriptor is consistent with the higher residual sugar content previously reported for the *S. boulardii* formulation by Pereira et al. (2025) [4], although the residual sugar content of the specific batches used in the present sensory evaluation was not independently quantified. These findings corroborate the results of the hedonic analysis, in which this same sample achieved higher scores for flavor, overall impression, and purchase intent, reinforcing the potential of *S. boulardii* to improve the sensory acceptance of cupuaçu melomel.

Figure 2 presents the radar chart of the sensory descriptors that showed significant differences in the Check-All-That-Apply (CATA) analysis of the cupuaçu melomels fermented with *S. boulardii* and *S. cerevisiae*. The chart provides a descriptive and visual representation of the frequencies of descriptors selected by consumers. Statistical differences between treatments were determined using Cochran’s Q test (*p* < 0.05), rather than by the visual separation of the polygons in the chart. The results indicate differences in the sensory profiles of the two treatments, showing that the melomels produced with the two yeast strains differed significantly in their sensory characterization.

As shown in Figure 2, the *S. boulardii* melomel was more frequently associated with “light amber color,” “clear,” “honey aroma,” “sweet taste,” “honey flavor,” and “Flavorful.” This highlights a sensory profile characterized by enhanced visual clarity, more intense honey aromatic notes, and a greater perception of sweetness and flavor intensity. Conversely, the *S. cerevisiae* fermented melomel showed a stronger association with the descriptors “cloudy,” “bitter taste,” “dry taste,” “vinegary flavor,” and “alcoholic taste,” indicating a sensory profile defined by attributes typically linked to lower consumer preference, particularly for fermented beverages intended for direct consumption.

Overall, the CATA analysis results corroborated the data obtained from the hedonic evaluation, in which the melomel fermented with *S. boulardii* achieved higher scores for flavor, overall impression, and purchase intent. The convergence between the descriptive and affective results reinforces the hypothesis that using this probiotic yeast favored the development of a more attractive sensory profile with greater potential for consumer acceptance.

### 3.3. Limitations of This Study

It should be emphasized, however, that the main objective of this study was to investigate the sensory acceptance and sensory profile of cupuaçu mead produced with *S. boulardii* and *S. cerevisiae*. The beverage formulation and fermentation procedure were standardized according to the methodology previously established by Pereira et al. (2025) [4], which included the physicochemical characterization of cupuaçu mead. In this study, significant differences in soluble solids (°Brix) were observed among the formulations, with higher values for the treatment with *S. boulardii*, while total titratable acidity did not differ significantly among the treatments. Pereira et al. (2025) [4] also reported a higher residual sugar content in the beverage fermented with *S. boulardii*.

It acknowledges, however, that the present study involved new batches prepared for sensory evaluation and that the physicochemical parameters of these specific batches were not determined independently. Therefore, it cannot be assumed that the values reported by Pereira et al. (2025) [4] are identical to those of the samples evaluated by consumers. That said, it should be clarified and interpreted that the sensory differences observed were not caused exclusively by microorganisms but were associated with the use of different yeast strains under standardized fermentation conditions. Thus, since yeast metabolism can affect residual sugars, ethanol, organic acids, volatile compounds, clarification, and other characteristics of the fermented matrix, differences in sensory perception may reflect the combined effects of each yeast’s specific metabolic activity and the resulting physicochemical composition of the beverages. We explicitly acknowledge the lack of physicochemical characterization of the specific lots used in the sensory evaluation as a limitation of this study, and we suggest that future studies should combine sensory evaluation with physicochemical characterization and, ideally, the characterization of the volatile compounds from the same lots, in order to establish more direct relationships between yeast metabolism, the composition of the beverage, and sensory perception.

Another limitation of this study relates to the demographic composition and prior familiarity of the consumer panel. Although the 113 consumers provided useful information for the initial screening of sensory acceptance and product differentiation, the panel was predominantly composed of young adults, with 72.6% of participants aged 18 to 24, and approximately 88.5% reported having no prior experience with mead. From a sensory science perspective, this group of consumers is valuable during the exploratory stages of product development, particularly for identifying overall acceptance, sensory attributes associated with preference, and possible differences among formulations. However, this sample profile may not comprehensively reflect the diversity of potential melomel consumer segments. Therefore, the results obtained should be interpreted in light of the characteristics of the sample studied; they cannot be generalized to the entire consumer population or considered, in isolation, as definitive evidence of the product’s market acceptance. Future studies should investigate more heterogeneous and market-oriented consumer groups, including consumers of different age groups, levels of familiarity with melomel, and patterns of alcoholic beverage consumption, in order to better characterize the target market and confirm the commercial potential of cupuaçu melomel.

## 4. Conclusions

This study investigated the sensory acceptance and sensory profile of cupuaçu mead produced with the probiotic yeast *S. boulardii* and the commercial yeast *S. cerevisiae*. The mead fermented with *S. boulardii* exhibited superior sensory performance compared to that produced with *S. cerevisiae*, achieving significantly higher scores for flavor, overall impression, and purchase intention. Although the melomel fermented with *S. boulardii* showed a significantly higher purchase intention than the formulation fermented with *S. cerevisiae*, the absolute difference was 0.54 points on the seven-point scale. This difference indicates a favorable consumer response to the formulation with *S. boulardii* but should be interpreted as an initial indication of market potential rather than as direct evidence of commercial success.

The CATA method allowed for the differentiation of the beverages’ sensory profiles, demonstrating that the formulation produced with *S. boulardii* was associated with desirable sensory attributes, including a light amber color, greater clarity, and a more pronounced honey aroma and flavor. In contrast, the mead produced with *S. cerevisiae* was associated with a more alcoholic, dry, and bitter profile, characteristics that negatively influenced consumer preference.

The results of this study demonstrated the potential for applying probiotic yeasts in the development of fermented alcoholic beverages with distinct sensory characteristics. However, further consumer and market studies involving larger and more representative populations are needed to confirm whether this sensory advantage translates into actual purchasing behavior.

## Figures and Tables

**Figure 1:**
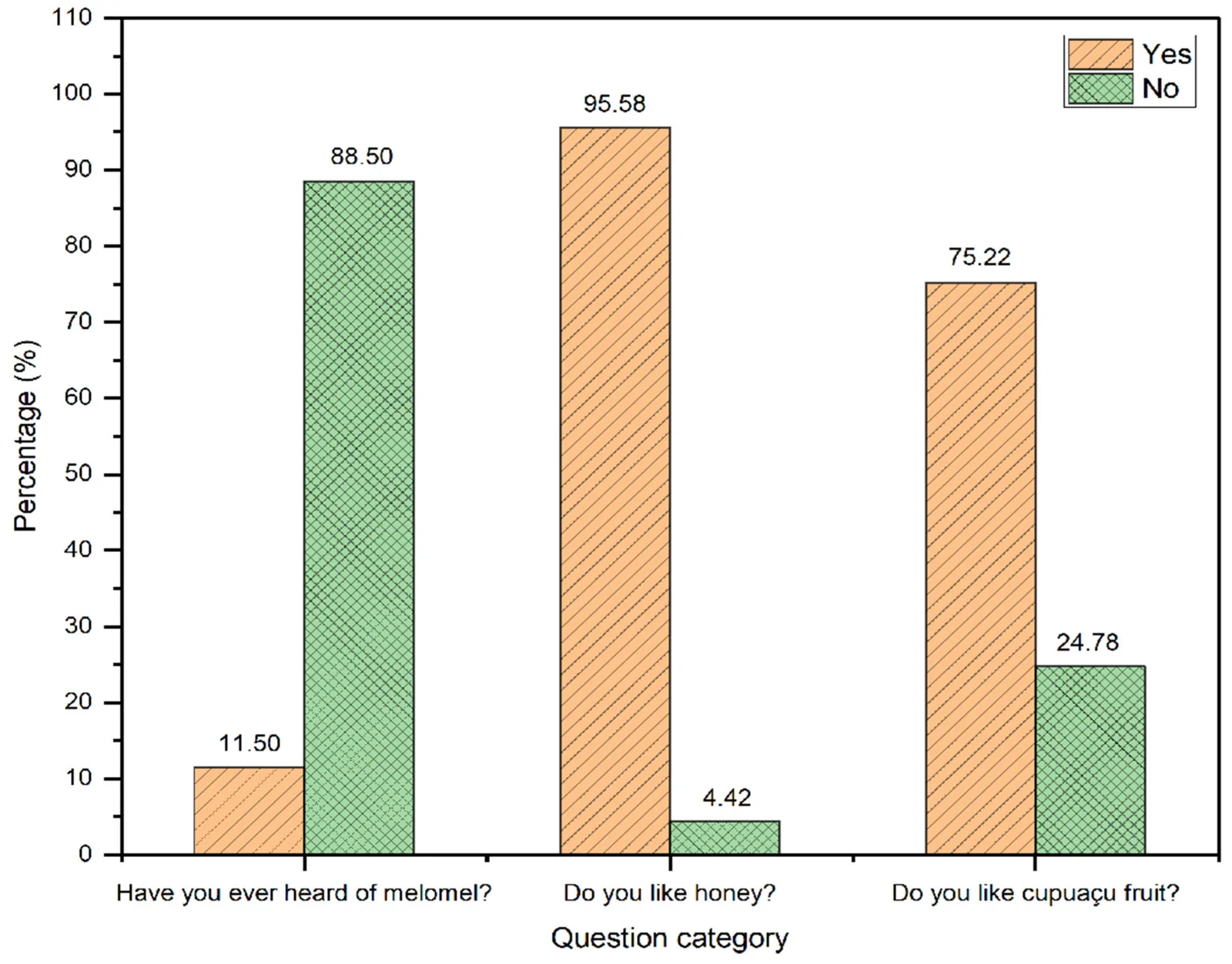
Familiarity with melomel and knowledge level about honey and cupuaçu between consumers who participated in sensory analysis of melomel (*N* = 113).

**Figure 2:**
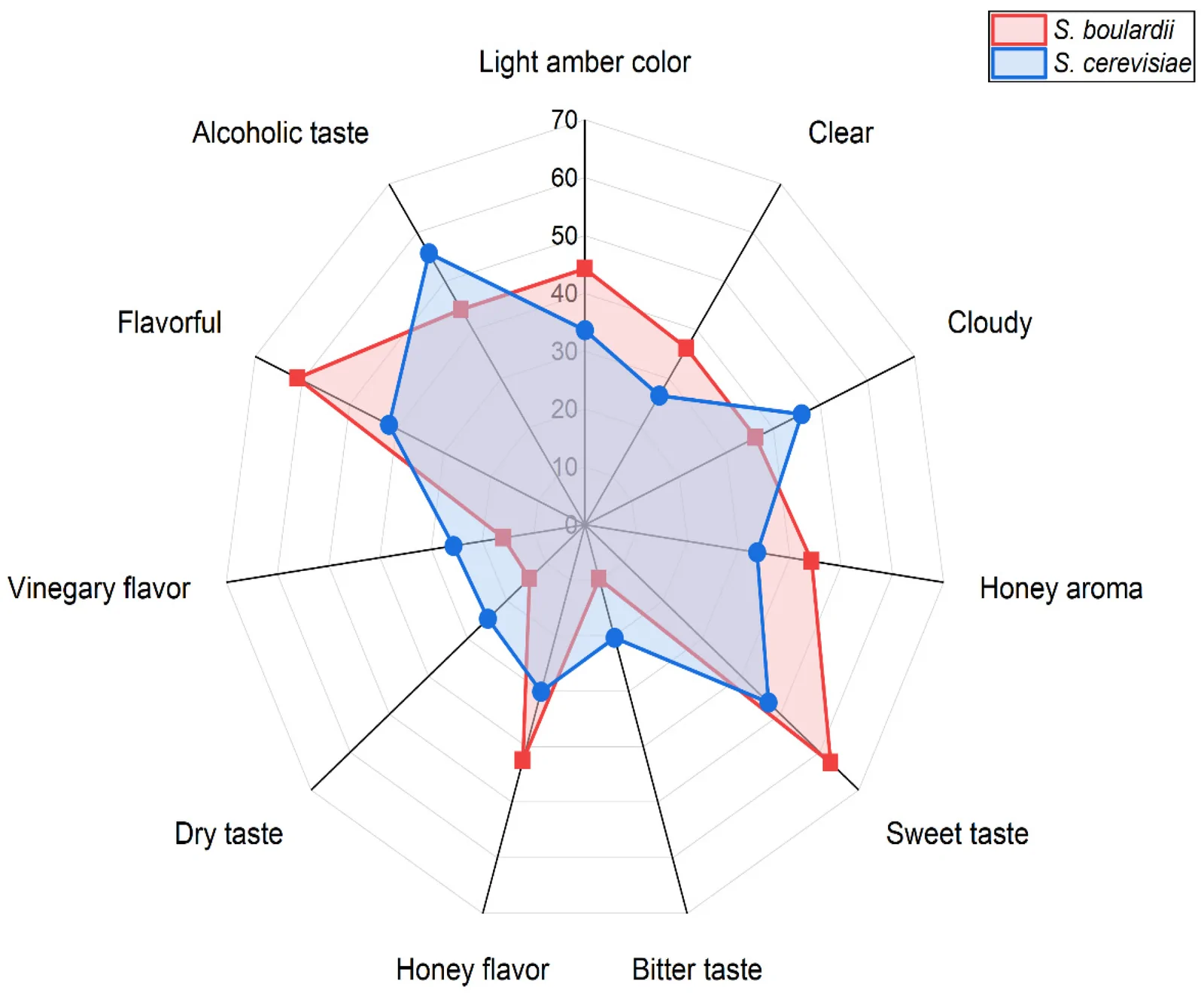
Radar chart of the significant sensory descriptors obtained from the Check-All-That-Apply (CATA) evaluation of cupuaçu melomel samples produced with *S. boulardii* and *S. cerevisiae*. Values represent the frequency (%) of consumers who selected each descriptor during the sensory evaluation (n = 113). The radar chart is presented for descriptive and visualization purposes, and statistical significance was determined using Cochran’s Q test (*p* < 0.05). Only descriptors showing significant differences are presented.

**Table 1:** Demographic characteristics and alcohol consumption frequency of consumers participating in the sensory evaluation of Cupuaçu melomel samples.

Demographics and Consumer Profile		Count (N = 113)	Percentage (%)
Gender	Female	75	66.4
	Male	38	33.6
Age Group (Years)	18–24	82	72.6
	25–29	17	15.0
	30–39	7	6.2
	40–49	5	4.4
	50–59	2	1.8
Alcohol consumption frequency	Daily	0	0
	Twice a week	39	34.5
	Four times a week	1	0.9
	Once a month	41	36.3
	Do not consume frequently	8	7.1
	Occasionally	24	21.2

Percentages were calculated based on the total number of participants (N = 113).

**Table 2:** Hedonic scale scores for sensory attributes and purchase intention of Cupuaçu melomel samples produced with *Saccharomyces cerevisiae* var*. boulardii* and *Saccharomyces cerevisiae*.

Attributes	Cupuaçu Melomel *(S. boulardii)*	Cupuaçu Melomel *(S. cerevisiae)*
Overall impression	7.51 ± 1.15 ^a^	7.25 ± 1.18 ^b^
Flavor	7.64 ± 1.30 ^a^	7.04 ± 1.52 ^b^
Color	7.12 ± 1.46 ^a^	7.08 ± 1.44 ^a^
Aroma	7.04 ± 1.51 ^a^	6.94 ± 1.43 ^a^
Appearance	7.26 ± 1.35 ^a^	7.23 ± 1.29 ^a^
Purchase intention	4.63 ± 1.39 ^a^	4.09 ± 1.46 ^b^

^a,b^ In each row, means with different superscript letters differ statistically (*p* < 0.05) between treatments.

**Table 3:** Frequency (absolute counts) and *p*-value of Cochran’s Q test for each descriptor of the Check-All-That-Apply (CATA) evaluation of Cupuaçu melomel samples produced with *Saccharomyces cerevisiae var. boulardii* and *Saccharomyces cerevisiae* (n = 113).

Descriptor	Cochran’s Q (*p*-Value)	Cupuaçu Melomel Sample	
		*S. boulardii*	*S. cerevisiae*
Yellowish color	0.2367	57 ^a^	64 ^a^
Light amber color	0.0339	50 ^a^	38 ^b^
Clear	0.0343	41 ^a^	30 ^b^
Cloudy	0.0343	41 ^b^	52 ^a^
Fruity aroma	0.3657	46 ^a^	40 ^a^
Honey aroma	0.0455	50 ^a^	38 ^b^
Cupuaçu aroma	1.0000	27 ^a^	27 ^a^
Alcoholic aroma	0.1936	62 ^a^	69 ^a^
Vinegary aroma	0.8185	13 ^a^	12 ^a^
Sweet taste	0.0094	71 ^a^	53 ^b^
Bitter taste	0.0047	11 ^b^	23 ^a^
Acidic taste	0.4349	42 ^a^	47 ^a^
Cupuaçu flavor	0.2971	23 ^a^	18 ^a^
Honey flavor	0.0196	48 ^a^	34 ^b^
Dry taste	0.0285	16 ^b^	28 ^a^
Vinegary flavor	0.0343	18 ^b^	29 ^a^
Full-bodied	0.2850	9 ^a^	13 ^a^
Flavorful	0.0019	69 ^a^	47 ^b^
Distinctive	0.5775	37 ^a^	40 ^a^
Alcoholic taste	0.0196	50 ^b^	63 ^a^

^a,b^ In the same row, descriptors with different superscript letters differ significantly at *p* < 0.05 by Cochran’s Q test.

## Data Availability

The original contributions presented in this study are included in the article. Further inquiries can be directed to the corresponding authors.
